# Molecular Characterization of Gene-Mediated Resistance and Susceptibility of ESKAPE Clinical Isolates to *Cistus monspeliensis* L. and *Cistus salviifolius* L. Extracts

**DOI:** 10.1155/2022/7467279

**Published:** 2022-09-27

**Authors:** Imane Zalegh, Mohammed Bourhia, Khalid Zerouali, Khalid Katfy, Kaotar Nayme, Farid Khallouki, Ihssane Benzaarate, Ahmad Mohammad Salamatullah, Abdulhakeem Alzahrani, Hiba-Allah Nafidi, Mohamed Akssira, Rajaa Ait Mhand

**Affiliations:** ^1^RU Microbiology, Biomolecules and Biotechnology, Laboratory of Chemistry-Physics and Biotechnologies of Biomolecules and Materials, FST Mohammedia, Hassan II University of Casablanca, Casablanca, Morocco; ^2^Higher Institute of Nursing Professions and Technical Health, Laayoune 70000, Morocco; ^3^Laboratory of Microbiology-CHU Ibn Rochd Casablanca, Casablanca, Morocco; ^4^Laboratory of Microbiology, Faculty of Medicine and Pharmacy, University Hassan II Casablanca, Casablanca, Morocco; ^5^Molecular Bacteriology Laboratory, Institute Pasteur Du Marocco, 1 Place Louis Pasteur, Casablanca 20360, Morocco; ^6^Biology Department, FSTE, University Moulay Ismail, BP. 609, Errachidia 52000, Morocco; ^7^Microbiology, Health and Environment Team Research, Department of Biology, Faculty of Sciences, Chouaib Doukkali University, Route Ben Maachou, El Jadida 24000, Morocco; ^8^Department of Food Science & Nutrition, College of Food and Agricultural Sciences, King Saud University, P.O. Box 2460, Riyadh 11451, Saudi Arabia; ^9^Department of Food Science, Faculty of Agricultural and Food Sciences, 2325 University Street Quebec City, Quebec City G1V 0A6, Canada

## Abstract

**Background:**

Multidrug resistance (MDR) and extensively drug-resistant (XDR) are now the biggest threats to human beings. Alternative antimicrobial regimens to conventional antibiotic paradigms are extensively searched. Although *Cistus* extracts have long been used for infections in traditional folk medicines around the world, their efficacy against resistant bacteria still needs to be elucidated. We aim to investigate the antibiotic susceptibility profiles of clinical strains *Enterococcus faecium*, *Staphylococcus aureus*, *Klebsiella pneumoniae*, *Acinetobacter baumannii*, *Pseudomonas aeruginosa*, and *Enterobacter cloacae* (acronym “ESKAPE”), and their resistance mechanisms by PCR, as well as their sensitivity to *C. monspeliensis* (CM) and *C. salviifolius* (CS) methanol extracts and their fractions.

**Methods:**

Antibiotic susceptibility profile and resistance mechanism were done by antibiogram and PCR. Fractions of CM and CS were obtained using maceration and Soxhlet; their antibacterial activities were evaluated by determining inhibition zone diameter (IZD), minimum inhibitory concentration (MIC), and minimum bactericidal concentration (MBC).

**Results:**

Results revealed that all strains were XDR except *S. aureus*, which was MDR. The PCR indicates the presence of gene-mediated resistance (*bla*_CTX-M_, *bla*_SHV_, *bla*_OXA-48_, *bla*_NDM_, *bla*_OXA-51_, *bla*_OXA-58_, *bla*_IMP_, *bla*_VIM_, and *bla*_mecA_). Also, maceration was slightly better for bioactivity preservation. Overall, the extracts of CM (IZD = 20 mm, MIC = 0.01 mg/mL) were more active than those of CS. All extracts inhibited MRSA (methicillin-resistant *Staphylococcus aureus*) and ERV (*Enterococcus faecium* Vancomycin-Resistant) with interesting MICs. The ethyl acetate fraction manifested great efficacy against all strains. Monoterpene hydrocarbons and sesquiterpenes oxygenated were the chemical classes of compounds dominating the analyzed fractions. Viridiflorol was the major compound in ethyl acetate fractions of 59.84% and 70.77% for CM and CS, respectively.

**Conclusions:**

The superior activity of extracts to conventional antibiotics was seen for the first time in the pathogens group, and their bactericidal effect could be a promising alternative for developing clinical antibacterial agents against MDR and XDR ESKAPE bacteria.

## 1. Introduction

Nosocomial infections (NIs) are a growing threat to human beings and a burden in developed and developing countries [1]. However, the rate of NIs is two- to three-fold higher in developing countries compared to Europe or the United States of America [2]. The prevalence of nosocomial bacterial infections in Morocco is highly dominant in the intensive care unit (ICU) than in other services [3]. The presence of multidrug-resistant (MDR), extensively drug-resistant (XDR), and pan-drug-resistant (PDR) bacteria limits and complicates therapy, causes mortality, prolongs hospitalization duration, and results in a significant-high cost for patients as well as hospitals [4]. According to the Infectious Disease Society of America (IDSA), a principal focus should be assigned to six pathogens referred to as “ESKAPE,” an acronym for *Enterococcus faecium*, *Staphylococcus aureus*, *Klebsiella pneumoniae*, *Acinetobacter baumannii*, *Pseudomonas aeruginosa*, and *Enterobacter cloacae*.

This group is perilous in clinical practice because of its potential multidrug resistance mechanisms to conventional antibiotics (ATB) and virulence [5]. The World Health Organization recognizes these MDR, XDR, and PDR bacteria as the leading causes of death in 2050 (more than 10 million deaths per year caused by antimicrobial resistance), which is more than cancer currently causes deaths [6].

In this context, finding new drugs and innovation strategies are urgently needed. Phytochemical research has received more attention as a potential source for new therapeutic compounds in the last decade due to their known biological functions since ancient times. Medicinal plant use and traditional medicine practices in East and Central Africa are still the predominant forms of healthcare [7]. Thus far, *Cistus* species have demonstrated a strong potential to fight pathogenic microorganisms such as viruses, fungi, parasites, and bacteria [8]. These properties were described to be associated with polyphenol compounds, such as diterpenes, oxygenated sesquiterpenoids, terpenoids, flavonoids, fatty acids, and hydrocarbons [8]. Furthermore, using some *Cistus* species as a food supplement and herbal tea is widespread worldwide, such as CYSTUS® by Dr. Pandalis. A recent ethnobotanical study by Bouyahya et al. reported that these species, locally called “touzal,” play a significant role in Moroccan traditional medicine, particularly for skin, wound infections, and treating symptoms associated with gastral disorders [9].

This study aimed to evaluate the antibiotic susceptibility profiles of 6 different clinical strains isolated from patients hospitalized in the University Center Hospital Ibn Rochd and determine their molecular mechanisms of resistance. The antibacterial activity of organic extracts of the two *Cistus* species: *Cistus salviifolius* (CS) and *Cistus monspeliensis* (CM), was also determined on these ESKAPE MDR strains, and the phytochemical composition was also performed by GC-MS analysis. As far as the authors are aware, no study was carried out in that context.

## 2. Materials and Methods

### 2.1. Plant Collection

In order to use autochthonous Moroccan specimens of *Cistus*, CM, and CS, plants were obtained from the natural park of the province of Benslimane (33.623024–7.108652) in May 2018. The authors identified specimens based on the morphology of leaves and flowers. Fresh aerial parts were processed independently, cleaned up to remove residues of dust and arthropods, then shade dried at room temperature for three months.

### 2.2. Extract Processing and Fractionation

**Figure 1:**
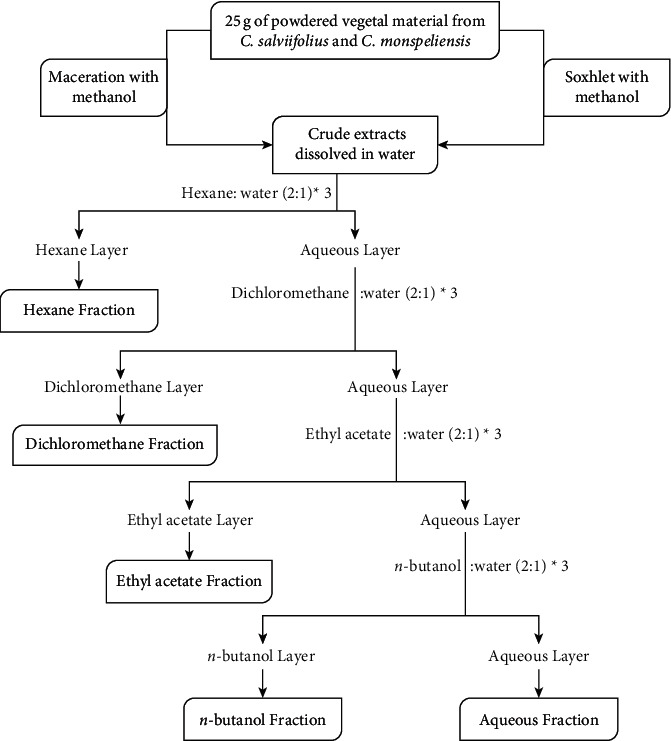
Scheme of extraction of plant material and fractionation with different solvents.

The samples were crushed with a grinder to obtain fine particles and stored in a hermetically sealed glass jar to avoid humidity and protected from light at ambient temperature (25°C). 25 g of powdered materials were either extracted by cold maceration (at room temperature for 72 h) or Soxhlet (8 h at a temperature no higher than 70°C). To selectively extract different compounds from the samples, extraction procedures were conducted using methanol. The filtered solutions were evaporated to dryness at 40°C using a rotary evaporator (crude extract 1 and 1′). The resulting residues of two *Cistus* species from both extraction techniques were dissolved in distilled water, and a typical fractionation scheme involves several steps, as illustrated in Figure 1, using the following solvents: hexane, dichloromethane, ethyl acetate, and *n*-butanol, yielding four fractions in addition to the remaining aqueous solution, which constituted fraction 5. For all fractions, solvents were removed in vacuo using a rotary evaporator. The 24 CM and CS crude extracts and their fractions were stored in a freezer at −20°C until further analysis.

### 2.3. Gas Chromatography-Mass Spectrometry (GS-MS) Analysis

The extracts of CS and CM were dissolved in hexane. The separation and identification were performed on a Shimadzu GC system (Kyoto, Japan) equipped with a BPX25 capillary column with 5% diphenyl and 95% dimethylpolysiloxane phase (30 m × 0.25 mm inner diameter × 0.25 *μ*m film thickness), coupled to a QP2010 MS. Pure helium gas (99.99%) was used as a carrier gas with a constant flow rate of 3 mL/min. The injection, ion source, and interface temperatures were all set at 250°C. The temperature program used for the column oven was 50°C (held for 1 min), heated to 250°C at 10°C/min, and held for 1 min. The ionization of the sample components was done in the EI mode (70 eV). The mass range scanned was 40–300 m/z. 1 *μ*L of each prepared extract diluted with an appropriate solvent was injected in a splitless mode (split ratio 90 : 1). All samples were analyzed in triplicate. Finally, compounds were identified by comparing their retention times with those of authentic standards and their mass spectral fragmentation patterns with those found in databases or those stored on the National Institute of Standards and Technology (NIST) 147, 198 compounds. LabSolutions (version 2.5) was used for data collection and processing.

### 2.4. Collection of ESKAPE Clinical Isolates and Identification

In this study, 6 ESKAPE strains: *Enterococcus faecium*, *Staphylococcus aureus*, *Klebsiella pneumoniae*, *Acinetobacter baumannii, Pseudomonas aeruginosa*, and *Enterobacter cloacae*, were collected from different samples at the Microbiology Laboratory in the Ibn Rochd University Hospital Center Casablanca Morocco. They were all identified according to conventional biochemical methods [10].

### 2.5. Antibiogram of Isolates

Antibiotics susceptibility testing of the selected strains was determined on Mueller–Hinton agar using the standard disk diffusion for the following antibiotics: ampicillin AMP (10 *μ*g), amoxicillin clavulanic acid AMC (20/10 *μ*g), piperacillin/tazobactam PTZ (30/6 *μ*g), cefalexin CFX (30 *μ*g), ceftriaxone CRO (30 *μ*g), cefotaxime CTX (5 *μ*g), ceftazidime CAZ (10 *μ*g), cefepime CPM (30 *μ*g), cefoxitin FOX (30 *μ*g), meropenem MEM (10 *μ*g), ertapenem ETP (10 *μ*g), imipenem IMP (10 *μ*g), gentamicin GM (30 *μ*g), ciprofloxacin CIP (5 *μ*g), levofloxacin LEV (5 *μ*g), amikacin AK (30 *μ*g), tobramycin TN (10 *μ*g), netilmicin NET(10 *μ*g), tigecycline TGC (15 *μ*g), trimethoprim/sulfamethoxazole TSU (1.25/23.75 *μ*g), cotrimoxazole SXT (25 *μ*g), kanamycin K (30 *μ*g), penicillin G PG (1 U), erythromycin E (15 *μ*g), linezolid LNZ (10 *μ*g), teicoplanin TEC (30 *μ*g), and vancomycin VA (5 *μ*g). The susceptibility to c olistin CL was determined by broth microdilution. Methods and interpretation of results were made according to the Clinical and Laboratory Standards Institute (CLSI) [11] and European Committee on Antimicrobial Susceptibility Testing (EUCAST) clinical breakpoints [12]. Phenotypic detection of ESBL production for *K. pneumoniae* and *E. cloacae* was detected by the double-disc synergy test [11].

### 2.6. Extraction of Genomic DNA

The extraction of genomic DNA was performed using the boiling method previously described by Honoré et al. [13]. Pure bacteria colonies from overnight cultures of each ESKAPE isolate growing on appropriate agar were suspended in 400 *μ*L of distilled water. The suspensions were boiled at 100°C for 10 min in a thermal block, then placed into an icebox for 3 min, and centrifuged at 12000*g* for 10 min. An aliquot of 180 *μ*L of the supernatant was used as a DNA template for PCR.

### 2.7. Detection of Gene-Mediated Resistance

To detect gene resistance in isolated strains by PCR amplification, we used the primers listed in Table 1.

*K. pneumoniae* and *E. cloacae* ESBL-producing were screened by PCR simplex for the following *β*-lactamase-encoding genes: *bla*_CTX-M_, *bla*_SHV_, and *bla*_TEM_, as described by Guessennd et al. [14]. Also screened by PCR for the following carbapenemase-encoding genes: *bla*_OXA-48_, *bla*_NDM_, and *bla*_VIM_, described by Dallenne et al. [15] with slight modifications. For *bla*_CTX-M_, *bla*_SHV_, and *bla*_TEM_, amplification mixture was performed in volume of 23.5 *μ*L containing: 2.5 *μ*L of MgCl_2_ (2.5 mM), 5 *μ*L of buffer, 0.5 *μ*L of each forward and reverse primers (0.2 *μ*M), 0.5 *μ*L of dNTP (0.2 mM), 14.3 *μ*L of ddH_2_O, and 0.2 *μ*L (1 unit) of Taq DNA polymerase. Then we added 1.5 *μ*L of the bacterial DNA for each simplex PCR.

For *bla*_OXA-48_, amplification mixture was performed in volume of 22 *μ*L containing: 2.5 *μ*L of MgCl_2_ (2.5 mM), 5 *μ*L of buffer, 0.5 *μ*L of each forward and reverse primers (0.2 *μ*M), 0.5 *μ*L of dNTP (0.2 mM), 12.8 *μ*L of ddH_2_O, and 0.2 *μ*L (1 unit) of Taq DNA polymerase. Then we added 3 *μ*L of the bacterial DNA. Amplification reactions for *bla*_NDM_ and *bla*_VIM_ were performed in a volume of 22 *μ*L containing: 2.5 *μ*L of MgCl_2_ (2.5 mM), 5 *μ*L of buffer, 1 *μ*L of each forward and reverse primers (0.2 *μ*M), 2 *μ*L of dNTP (0.2 mM), 10.3 *μ*L of ddH_2_O, and 0.2 *μ*L (1 unit) of Taq DNA polymerase. Then we added 3 *μ*L of the bacterial DNA.

*A. baumannii* resistant to imipenem was screened by simplex real-time for the following carbapenemase-encoding genes: *bla*_OXA-51_, *bla*_OXA-23_, *bla*_OXA-58_, *bla*_VIM_, *bla*_IMP_, *bla*_NDM_, and *bla*_KPC_. Amplification reactions for those genes were performed in a volume of 15 *μ*L containing: 10 *μ*L of SensiFAST SYBR NO-ROX Mix, 0.8 *μ*L of each forward and reverse primers (400 nM), 3.4 *μ*L of ddH_2_O. Then we added 5 *μ*L of the bacterial DNA. *P. aeruginosa* resistant to imipenem was screened for the following carbapenemase-encoding genes: *bla*_NDM_, *bla*_VIM_, *bla*_IMP_, *bla*_KPC_, and *bla*_OXA-48_. Amplification reactions for the used genes were performed in a volume of 24 *μ*L, containing 2.5 *μ*L of MgCl_2_ (2.5 mM), 5 *μ*L of buffer, 1 *μ*L of each forward and reverse primers (0.4 *μ*M), 0.5 *μ*L of dNTP (100 *μ*M), 13.6 *μ*L of ddH_2_O, and 0.4 *μ*L (2 unit) of Taq DNA polymerase. Then we added 2 *μ*L of the bacterial DNA. PCR cycling conditions for genes screened for *P. aeruginosa* are summarized in Table 2.

The MRSA isolate was screened for the methicillin-encoding gene *bla*_mecA_ and the vancomycin-encoding gene *bla*_VanA_ as described [16]. For *bla*_mecA,_ amplification mixture was performed in a volume of 25 *μ*L containing: 1 *μ*L of the bacterial DNA and 1 *μ*L of each forward and reverse primers (0.4 *μ*M), 19.5 *μ*L of ddH_2_O, 2.5 *μ*L of MyTaq Bioline (Buffer, dNTP, MgCl_2_ and Taq DNA polymerase). For *bla*_VanA,_ amplification mixture was performed in a volume of 48 *μ*L containing: 1.25 *μ*L of MgCl_2_ (1.25 mM), 5 *μ*L of buffer and 1 *μ*L of each forward and reverse primers (0.4 *μ*M), 0.625 *μ*L of dNTP (0.125 mM), 38.725 *μ*L of ddH_2_O, and 0.4 *μ*L (2 unit/*μ*L) of Taq DNA polymerase. Then 2 *μ*L of the bacterial DNA was added to the mixture. The amplification was done using Applied Biosystems by Life Technology 2720ThermoCycler machine. PCR cycling conditions for all genes are summarized in Table 3.

Amplicons were visualized after running at 120 V for 30 min on a 0.5% agarose gel containing ethidium bromide (0.5 *μ*g/mL) using a photographed UV transilluminator and analyzed compared with the positive control of each resistance gene and DNA ladder 100 bp (Promega).

### 2.8. Antibacterial Effect of Extracts

The antibacterial activity of the 24 *Cistus* extracts obtained by maceration and Soxhlet methods (12 of CS and 12 of CM) was evaluated by disc diffusion and microdilution methods by determining minimum inhibitory concentration (MIC) and minimum bactericidal concentration (MBC). MIC was considered as the lowest concentration of the extract preventing visible growth, and MBC was recorded as the lowest extract concentration killing 99.9% of the bacterial inoculate.

#### 2.8.1. Screening by Disc Diffusion

All extracts were initially screened by the disc diffusion method, following the standard protocol M02-A11 from the CLSI [17]. The dried plants' extracts were dissolved in dimethylsulphoxide (DMSO) and homogenized using ultrasound apparatus. *K. pneumoniae*, *E. cloacae*, *P. aeruginosa*, and *A. baumannii* were grown on MacConkey agar, *S. aureus* on Chapman agar, and *E. faecium* on Bile Esculin agar. Isolated colonies from each bacterium were transferred to tubes containing sterile distilled water to reach 0.5 McFarland turbidity (equivalent to 10^8^ CFU/mL) as a stock solution. For the tests, a dilution (1/100) was prepared in sterile conditions and used for final inoculum concentrations (10^5^–10^6^ CFU/mL). After that, Mueller-Hinton agar plates were inoculated with the bacterial inoculum spread. 10 *μ*L of each extract were placed on 6 mm diameter sterile paper discs (Whatman No.3). The standard drugs for comparison were limited because of the high resistance of selected strains we used: gentamicin GM (10 *μ*g) and tigecycline. One disc with DMSO as a negative control was placed on each plate. Then, the agar plates were incubated at 37°C for 24 h. The inhibitory zone diameter (IZD) was measured after incubation in millimeters. The experiment was carried out in three independent replicates.

#### 2.8.2. Determination of Minimal Inhibitory Concentration (MIC)

MIC determination of *Cistus* extracts against the selected strains was performed in a U-bottom 96-well microplate, using a modified microdilution protocol previously described by CLSI guidelines [11, 18]. For the assay, from a stock solution of extracts dissolved in DMSO, final extract concentrations were made based on the screening of agar diffusion results, ranging from 27.5 to 0.01 mg/mL. The standard inoculum was prepared in sterile distilled water from fresh culture colonies of the selected bacteria at an optical turbidity of 0.5 McFarland. Subsequently, dilution was made to normalize a final bacterial population of 10^5^ CFU/mL in each well. The following controls were used: culture medium control and growth control. Finally, microplates were incubated at 37°C for 24 h.

Thus, 25 *μ*L of 2,3,5-triphenyltetrazolium chloride (TTC) aqueous solution (1%) was added to each well. After further incubation for one hour at 37°C, visual inspection of viable cells was evidenced by producing a red color [19]. The MICs were established as the lowest extract concentration that inhibited visible bacterial growth.

#### 2.8.3. Determination of Minimal Bactericidal Concentration (MBC)

The determination of MBC was performed from wells containing extract concentrations without any visible bacterial growth. 10 *μ*L from each well was subcultured on nutrient agar in Petri dishes, which were incubated for 24 h at 37°C. The MBC was defined as the lowest concentration of extracts that resulted in >99% bacterial inactivation from the initial bacterial inoculum; for our case, it was defined as the absence of visible colonies on the agar plates after reincubation.

Three technical replicates were performed for each individual assay. Moreover, for each extract, the ratio of MBC/MIC was calculated to determine the type of effect of *Cistus* species. The extract has a bactericidal effect when the ratio is  ≤4 and a bacteriostatic effect if the ratio is  >4 [20].

## 3. Results

### 3.1. Yields of Crude Extract and Fractions

The aerial parts of CS and CM were extracted using cold maceration and Soxhlet extraction in methanol and fractionated with hexane, dichloromethane, ethyl acetate, and *n*-butanol, as described in the material section. These procedures yielded 24 different extracts, which were green or brown and aromatized. Details on obtained yields are shown in Table 4, which shows that yields varied significantly from 0.7 to 56.66%, depending on the extraction method and solvent. We have noted that the highest yield percentage was recovered from CM by methanol with Soxhlet (56.66%).

### 3.2. GC-MS Analysis of *C. monspeliensis* and *C. salviifolius* Fractions

**Figure 2:**
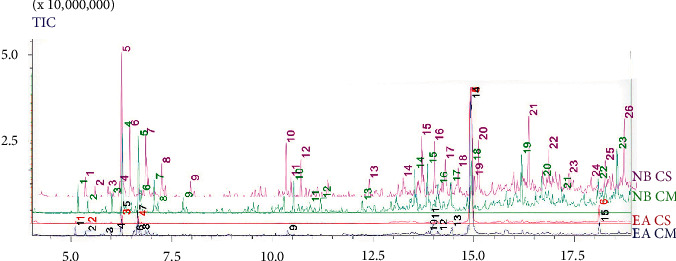
GC-MS peak chromatograms of 4 *Cistus* fractions: EA CM for ethyl acetate from *C monspeliensis*, EA CS for ethyl acetate from *C salviifolius*, NB CM for *n*-butanol from C monspeliensis, and NB CS for *n*-butanol from *C salviifolius*.

The chemical composition of ethyl acetate and *n*-butanol fractions of *C. monspeliesis* and *C. salviifolius* was performed by GC-MS analysis.  Table 5 represents the identified compounds, and Figure 2 represents GC-MS peak chromatograms of 4 *Cistus* fractions. In general, monoterpenes and sesquiterpenes were the chemical classes of compounds dominating the analyzed fractions, with other nonterpene volatile compounds such as hydrocarbons, alcohols, acid derivatives, ketones, esters, and ether also being identified.

A total of 31 and 24 compounds were found in *C. monspeliensis and C. salviifolius,* respectively. The major groups were found to be sesquiterpenes oxygenated (61.44% for CM, and 70.77% for CS), more abundant in the ethyl acetate fractions; monoterpene hydrocarbons (21.15% for CM, and 16.93% for CS); as well as nonterpene hydrocarbons (36.81% for CM, and 44.75% for CS), which were present in *n*-butanol fractions.

Among ethyl acetate's compounds, the most abundant one was viridiflorol, with 70.77% and 59.84% for CS and CM, respectively. *α*-pinene, *β*-myrcene, camphene, *α*-phellandrene, and limonene were identified in both species; however, the percentages were slightly higher in CM. Furthermore, *β*-pinene (1.85%) and cedrene (3.89%) were only detected in CM, while 1,1-dibutoxy-butane (3.56%) and agarospirol (2.49%) were present only in *C. salviifolius*. On the other hand, diterpene hydrocarbons appeared only in the *n*-butanol fractions, with lower intensity in CS (1.51%) than in CM (12.83%). In contrast, 2,6,10,14,18-pentamethyl-eicosane was identified in n-butanol CS's fraction (8.09%) compared to CM's *n*-butanol fraction (5.45%). Besides the compounds mentioned above, many others with relatively high area values were identified, such as elemol, 2,4-bis(1,1-dimethylethyl)-phenol, 2-ethyl-2-methyl-tridecanol, and 4-acetyl benzoic acid (5.43%, 5.77%, 4.72%, and 4.33% in CM respectively) and butyl butyrate,8-methyl-heptadecane, and 8-hexyl-pentadecane (13.6%, 8.08% and 6.51% in CS).

### 3.3. Antibiogram and Molecular Resistance Analysis of Isolates

Overall antibiotic susceptibility testing data showed that all isolates were highly resistant to the most antibiotics tested (Table 6). The phenotypical confirmatory test for production of ESBL was positive for the two Enterobacteriaceae: *K. pneumoniae* and *E. cloacae*. *A. baumannii* and *P. aeruginosa* were resistant to imipenem, *S. aureus* was resistant to methicillin (MRSA), and *E. faecium* was vancomycin-resistant (ERV). For instance, *S. aureus* was classed as MDR, *K. pneumoniae*, *E. cloacae*, *P. aeruginosa*, *E. faecium*, and *A. baumannii* were classed as XDR.

Molecular screening resistance genes were exanimated by PCR and showed that all isolates harbored genetic support of the resistance. In *K. pneumoniae* and *E. cloacae*, there is a combination of ESBLs *bla*_CTX-M_ or *bla*_SHV_ and carbapenemases *bla*_OXA-48_. *A. baumannii* was confirmed by detecting *bla*_OXA-51_ and producing more than one carbapenemase: *bla*_OXA-58_, *bla*_IMP_, and *bla*_NDM_. *bla*_NDM_ was detected in *P. aeruginosa*, while *bla*_mecA_ and *bla*_VanA_ encoded the resistance to Methicillin and Vancomycin in *S. aureus* and *E. faecium*.

### 3.4. Antibacterial Activity of *Cistus* Extracts

This study is the first one that evaluates the potential of CS and CM crude extracts and their fractions by using two methods (maceration and Soxhlet extraction) on XDR ESKAPE pathogens. The results of the antibacterial activity screening of the twenty-four *Cistus* extracts are shown in Tables 7 and 8.

By the disc diffusion method, all strains present different degrees of sensitivity (weak to high) with most extracts tested. IZD ranged from 6 to 20 mm for CM and 6 to 16 mm for CS. It is important to note that, in general, the two *Cistus* species showed very similar activity towards the same bacterial species. However, slightly better activity was demonstrated with the species *C. monspeliensis*. Also, MRSA, ERV, and *P. aeruginosa* imipenem resistant were the most sensitive to all the extracts tested.

On the other hand, MIC values obtained in our experiments ranged from 0.01 to 27.5 mg/mL. MIC and MBC values were reported in Tables 7 and 8. Extracts of both species obtained with methanol maceration were more active against tested strains (MIC = 0.01 to 3.43 mg/mL) than those obtained with Soxhlet (MIC = 0.05 to 13, 75 mg/mL). Macerated methanol extract of CM was less active (MIC = 0.01 to 3.43 mg/mL) than the relatively polar fractions (ethyl acetate), which was the most active against all strains, especially against *P. aeruginosa* imipenem resistant (MIC = 0.02 mg/mL). In addition, *K. pneumoniae* was very sensitive to this fraction (MIC = 0.42 mg/mL) compared to the other extracts (MIC = 1.71 to 6.87 mg/mL).

Among all the ESKAPE pathogens tested, only MRSA and ERV were inhibited by all the extracts tested with interesting MICs. The methanolic extract of CS inhibited MRSA with MIC = 0.10 mg/mL. However, extracts derived from Soxhlet methanol extract exhibited lower activity (MIC = 0.01 to 3.43 mg/mL) than macerated extracts derived from MIC = 0.01 mg/mL. Likewise, CM extract was strongly active against ERV (MIC = 0.42 mg/mL).

Overall, CM and CS extracts presented the lowest differences between MIC and MBC values. Thus, again, both species showed the best bactericidal activity against all XDR strains. However, a bacteriostatic effect was shown against MRSA.

## 4. Discussion

Plant-derived products play an essential role in finding biomolecules to treat infectious diseases that cause a global challenge for clinicians. The current study elucidates the molecular mechanisms of resistance of MDR and XDR ESKAPE pathogens. Furthermore, it presents and compares the antibacterial potentials of various extracts derived from the areal parts of *C. monspeliensis* and *C. salviifolius* against ESKAPE isolates.

Based on the current investigation, we have noted that methanol Soxhlet extraction provides the plant's highest extractive amount of secondary metabolites. Because heat increases solubility, diffusivity coefficient, and morphological changes in the plant sample matrix [21, 22], a polar solvent such as methanol is well known to be more effective in extracting bioactive compounds from plant materials [23].

Consequently, these parameters increase the rate of extraction. However, the yield of CS methanol extract obtained with maceration (27.9%) was compared to that reported by El Euch et al., who found 21.75% and 30.20% were obtained from leaves and flower bunds [24].

Fractionation of crude extracts and increasing polarity solvents in both techniques depends mainly on the analytes' solubility and their interactions with other constituents related to their structures [25, 26]. The results indicate that nonpolar solvents such as hexane showed low capability for extracting bioactive compounds; effectively, the yield recovered was 0.7% to 1.5%. These differences may be attributable to the higher solubility of extractable phytochemical components in polar solvents.

As far as the authors are aware, no phytochemical analysis was conducted on fractions from *C. monspeliensis* and *C. salviifolius* crude extracts. Most studies were conducted on essential oils (EO) and a few on crude extracts. Thus, no representative comparison could be made. However, regarding the qualitative presence of the phytoconstituents (monoterpenes, sesquiterpenes, diterpenes, nonterpene hydrocarbons, phenolic constituents, and the other groups) in the analyzed fractions and the published data from these *Cistus* species, and from other species such as *C. ladaniferus*, *C. villosus*, *C. libanotis*, etc., reveals similarities [8]. Nevertheless, the quantitative analysis showed differences.

For *C. monspeliensis*, the EO from Tunisia and Croatia was dominated by diterpenes (38.1% and 48.2%, respectively), while this group was not detected in these fractions [27, 28]. In contrast, hydrocarbons were less (11.3% and 3.6%) and alcohols were not detected in Croatian EO, which were 36.81% and 6.74%, respectively, in the n-butanol fraction. The high quantity of viridiflorol (59.84%) in the ethyl acetate fraction is noteworthy, which is absent in the mentioned EO [27, 28]. Also, the amount of viridiflorol was lower in the hexane (nonpolar solvent) extract of Tunisian *C. monspeliensis* (1.4%) [29]. In addition, the hexane extract analyzed by [29]was marked by the dominance of fatty acids (43.3%) that were only 1.22% in the ethyl acetate fraction (polar solvent). The absence of *α*-pinene, *β*-pinene, *β*-myrcene, camphene, *α*-phellandrene, and limonene indicated that these compounds were also present in a good amount in the *C. monspeliensis* analyzed fractions.

The two fractions from *C. salviifolius* presented the main difference, that *n*-butanol contained a variety of compounds monoterpene (hydrocarbons 16.93% and oxygenated 5.4%) represented mainly by limonene (6.70%), *α*-phellandrene (5.97%), and 1,1-dibutoxy-butane (3.56%), also the high presence of hydrocarbons (44.75%) and a moderate amount of phenolic compounds (5%). On the other hand, ethyl acetate fraction presented a less variable profile; it was dominated by viridiflorol 70.77%, 4-acetyl benzoic acid 3.90%, and monoterpene hydrocarbon 3.8%. The abundance of sesquiterpene is in agreement with the published phytochemical profiles of *C. salviifolius* EO from Italy [27], Spain [30], and the Hashemite Kingdom of Jordan [31]. However, viridiflorol was absent or present in low quantity in these studies (4.6% EO from Italy), which revealed the abundance of other compounds such as camphor (43.86%), eucalyptol (19.14%) [30], germacrene D (9.1%) [27], E-ethyl cinnamate (17.5%), and manoyl oxide (13.2%) [31].

In light of the obtained results of plants belonging to the same genus, the presence and concentration of various constituents in extracts are not only species and biotic/abiotic conditions dependent, but also depend on the part of the plant studied, the type of extract, method of extraction, and solvent used. Orabi et al. have presented the chemical differences between flowers and leaves of *C. salviifolius* using two methods for extracting volatile compounds [31]. Furthermore, Menor et al. demonstrated the effect of drying methods and seasonal influence on the polyphenolic content in aqueous extracts from *C. salviifolius* [32]. Additionally, the fractionation procedure could explain the phytochemical differences, which suggests the presence of the other constituents in the other fractions.

Over the years, antimicrobial resistance has continued to reach alarming levels, particularly in the ESKAPE group. We have noted the presence of genes responsible for phenotypical resistance. Our findings agree with numerous studies that have reported the high resistance in the ESKAPE pathogens [33–38]. Despite pressure selection, especially in the ICU, XDR character is expected because of chromosomally encoded and acquired resistance genes by pathogens like *A. baumannii* and *P. aeruginosa*.

Obviously, we cannot report an epidemiological profile in the current study. However, international literature reports the global dissemination of the CTX-M enzyme [39, 40]. Also, the study of Barguigua et al., which was carried out on clinical isolates from the same Ibn Rochd university hospital center, reported the occurrence of *bla*_OXA-48_, suggesting a similar trend in our study [41]. The acquired carbapenem resistance in *A. baumannii* is often attributed to *bla*_OXA-23_ and *bla*_OXA-24_, which are predominant in some Moroccan studies [42–44]. However, the weak prevalence of *bla*_OXA-58_, *bla*_VIM_, and *bla*_IMP_ reported by many studies [43, 45], which were present in tested isolates, could be alarming since they could be easily transferred to other bacterial species. Furthermore, the *P. aeruginosa* harboring *bla*_NDM_ found, to the best of our knowledge, for the first time poses a clinical challenge due to its potential transferability.

It is well known that *S. aureus* is recognized as a significant pathogen of hospital-acquired infection. Also, the epidemiology of MRSA has been dynamic. In an era of rapid dissemination of genes-mediated resistance, in recent years, data have shown an increased acquisition of different staphylococcal chromosomal cassette (SCC) mec types around the world [46–49]. The vancomycin gene, vanA, was not observed in this strain. Nevertheless, vanA resistance has been reported in *S. aureus* isolates in many countries, including the USA [50, 51], India [52], Iran [53], Pakistan [54], Brazil [55], and Portugal [56].

Among ESKAPE pathogens, high frequencies of multidrug-resistant by harboring diverse resistance mechanisms limited the treatment of patients considerably and were associated with the highest mortality risk. The development of new approaches is an important therapeutic challenge.

In the current research, the results of the antibacterial activity on tested ESKAPE showed significant efficiency. So far, IZD is coherent with some literature reports on *Cistus* species extracts [57–59]. Based on the low MIC values obtained in our experiments, it appears that the diffusion method may not always be a reliable method for screening the antimicrobial activity of plant extracts. It is well known that the absence of an inhibition zone does not necessarily mean that the compounds are inactive, especially for the less polar compounds, which diffuse more slowly into the culture medium [60]. The diffusion assay is not suited to natural antimicrobial compounds that are scarcely soluble or insoluble in water. Thus, their hydrophobic nature prevents uniform diffusion through the agar medium [61]. Because some compounds could not diffuse well on agar, this could affect their activity and results, and this was supported by the lowest MIC obtained with the same extracts on the same pathogens panel. We also noted differences in activity comparing extracts from two extraction methods. This difference might be due to the possible thermal degradation of compounds caused by temperature and long extraction time by the Soxhlet method.

Bioassay-guided fractionation is an effective procedure to discover novel potential agents via obtaining active fractions. Using this approach, we reported the best activity with an ethyl acetate fraction. This result supported the fact that the active compounds are concentrated more in this fraction; this agrees with the observation of Mastino et al., who reported that ethyl acetate extract showed the highest inhibitory activity against *S. aureus* (MIC = 1.25 mg/mL) [62]. On the other hand, the MIC methanolic extract of CS obtained against MRSA was the lowest compared to a previous study that reported a MIC of 4 mg/mL against a clinical strain of *S. aureus* [63]. Likewise, the activity of CM on ERV was stronger than that reported with *Enterococcus faecalis* ATCC 29212 (MIC = 5 mg/mL) demonstrated by Hickl et al. [64]. At the same time, it is well known, as reported in many previous studies, that Gram-positive strains are more sensitive than Gram-negative, which are more resistant to antibacterial compounds due to the morphological difference and, above all, to the difference in the permeability of the cell wall [65, 66]. Our finding is quite interesting since MIC values for XDR strains were the lowest compared to the ATCC strains (data not shown). Also, regarding CM, MICs for XDR strains were lower compared to those reported for ATCC strains by Bouamama et al. [67]. Furthermore, hexane fraction and methanol extract could inhibit *E. cloacae* and *S. aureus* MDR with MIC ranging from 0.01 to 3.43 mg/mL. In contrast, the same extracts did not show any inhibition activity in the study of [68]. Similarly, all extracts of CS possess excellent antibacterial activity, more than the MICs described by Rebaya et al., who reported 3.125 mg/mL against standard *S. aureus* as a strong inhibition [69]. Most recently, the study concerned the activity of the aqueous extract against three clinical MDR strains was reported by Carev et al.; this study found MICs higher than those seen with our XDR strains [70].

As a matter of fact, no conclusive comparison could be made between our results and previous studies, depending mainly on the difference in antibiotic sensitivity profiles of the strains used.

Another finding of this current study was the bactericidal effect against all XDR strains except for MRSA. This may be related to hydrocarbon compounds (highly present in the analyzed fractions), which seem to disturb the ATPase efficiency or the proton mortice force. Thus, it decreases ATP quantity in the intracellular medium and prevents cell division and, therefore, the exponential growth of cells as described by Guinoiseau et al. [71]. Phytochemical investigations reported in this study and literature data for this species identified diterpenes, sesquiterpene oxygenated, terpenoids, flavonoids, fatty acids, and hydrocarbons [27, 29, 72, 73]. Since CM extracts are mainly composed of diterpenes and CS by sesquiterpenes, 13-epi-manoyl oxide, camphor, and viridiflorol are likely compounds responsible for the antibacterial activity shown [74, 75]. However, it should be noted that the inhibitory effects observed with natural extracts are generally a combination of multiple compounds that lead to several different action mechanisms. On the other hand, high polyphenols are not always correlated with antibacterial activity, as [32] demonstrated, which supports our finding for ethyl acetate and n-butanol fractions, where polyphenols were not highly detected. At the same time, our results support many traditional medicines, such as applications against wounds, respiratory disorders, diarrhea, and others [9, 76].

## 5. Conclusions

In conclusion, knowledge of the emergence and rapid spread of molecular epidemiology of resistance mechanisms in the ESKAPE group is becoming a global challenge in the therapeutic protocols for clinicians, especially with patients infected with XDR pathogens. Hence, it is becoming increasingly important to consider all possible new and perhaps old treatment sources.

According to our findings, we can affirm the outstanding and encouraging antibacterial potency of bioactive molecules present in CM and CS extracts studied for the first time against XDR ESKAPE strains. These *Cistus* extracts acted differently for each strain; the chemical analysis of the most active fraction revealed a variety of phytochemical groups, whose abundance were variants depending on the fraction. The ethyl acetate fractions were dominated by sesquiterpenes oxygenated, represented by viridiflorol as a major compound, while the n-butanol fractions were dominated by monoterpenes, diterpenes, and hydrocarbons. However, it may be worthwhile to investigate the chemical composition of the other fractions to establish the chemical profiles of the studied species.

Emphasizing their potency to inhibit bacterial growth and based on the traditional therapeutic uses, caution is required when interpreting the presented evidence. The *in vitro* results clearly do not reflect the complex interactions and effectiveness *in vivo*; thus, the studied extracts cannot replace synthetic medicine yet. Therefore, further phytochemical and pharmacological research needs to be carried out to confirm the current results, investigate their toxicity, and determine the mode of action responsible for the bactericidal activity. Hence, it appears that *C. monspeliensis* and *C. salviifolius* are potential candidates as growth-inhibiting agents, and this knowledge could be translated into likely active principles on XDR ESKAPE infections.

## Figures and Tables

**Table 1:** Primers for PCR amplification of gene-mediated resistance.

Genes	Primers	Sequences (5′–3′)	Products size (pb)	References
*bla*_CTX-M_	CTX–M1–F CTX–M1–R	TTGGTGACGATTTTAGCCGC GGT TAA AAA ATC ACT GCG TC	864	[77]
*bla*_SHV_	SHV–F SHV–R	TTATCTCCCTGTTAGCCACC GATTTGCTGATTTCGCTCGG	870	[78]
*bla*_TEM_	TEM–F TEM–R	ATA AAA TTC TTG AAG ACG AAA GAC AGT TAC CAA TGC TTA ATC A	1080	[14]
*bla*_OXA_	OXA–48–F OXA–48–R	GCTTGATCGCCCTCGATT GATTTGCTCCGTGGCCGAAA	281	[15]
*bla*_NDM_	NDM–F NDM–R	GGTTTGGCGTCTGGTTTTC CGGAATGGCTCATCACGATC	621	[79]
*bla*_VIM_	VIM–F VIM–R	GATGGTGTTTGGTCGCATA CGAATGCGCAGCACCAG	390	[79]
*bla*_OXA_	OXA–51–F OXA–51–R	TAATGCTTTGATCGGCCTTG TGGATTGCACTTCATCTTGG	353	[80]
*bla*_OXA_	OXA–23–F OXA–23–R	GATCGGATTGGAGAACCAGA ATTTCTGACCGCATTTCCAT	501	[80]
*bla*_OXA_	OXA–58–F OXA–58–R	AAGTATTGGGGCTTGTGCTG CCCCTCTGCGCTCTACATAC	599	[80]
*bla*_IMP_	IMP–F IMP–R	CTACCGCAGCAGAGTCTTTG AACCAGTTTTGCCTTACCAT	587	[81]
*bla*_VIM′_	VIM′–F VIM′–R	GGTGTTTGGTCGCATATCGCAAC TGTGCTKGAGCAAKTCYAGACCG	390	[82]
*bla*_IMP′_	IMP1–F IMP1–R	AGCAAGTTATCTGTATTCTT TTTRCTTTCDTTNARYCCTT	713	[83]
*bla*_NDM′_	NDM′–F NDM′–R	AATGGAATTGCCCAATAT CGAAAGTCAGGCTGTGTT	489	[83]
*bla*_OXA′_	OXA′–48–F OXA′–48–R	TTGGTGGCATCGATTATCGG GAGCACTTCTTTTGTGATGGC	744	[84]
*bla*_KPC′_	KPC′–F KPC′–R	ATGTCACTGTATCGCCGTCT TTACTGCCCGTTGACGCCCA	881	[83]
*bla*_mecA_	mec–A–F mec–A–R	GATATCGAGGCCCGTGGATT ACGTCGAACTTGAGCTGTTA	642	[85]
*bla*_VanA_	van–A–F van–A–R	GGGAAAACGACAATTGC GTACAATGCGGCCGTTA	732	[16]

**Table 2:** Amplification conditions of genes NDM, VIM, IMP, KPC, OXA-48, screened for *P. aeruginosa*.

Amplification steps	Temperature conditions/duration				
	*bla *_NDM_	*bla *_VIM_	*bla *_IMP_	*bla *_KPC_	*bla *_OXA-48_
Initial denaturation	94°C/5 min				
Denaturation	94°C/1 min				
Annealing	57°C/1 min	60°C/1 min	50°C/1 min	60°C/1 min	58°C/1 min
Extension	72°C/1 min				
Final elongation	72°C/7 min				
Number of cycles	30				

**Table 3:** Amplification conditions of genes CTX-M, TEM, SHV, OXA-48, NDM, VIM, OXA-51, OXA-23, IMP, mecA, and VanA, screened for Enterobacteriaceae, *A. baumannii*, *S. aureus*, and *E. faecium*.

Amplification steps	Temperature conditions/duration						
	*bla *_CTX-M_, _SHV_	*bla *_TEM_	*bla *_OXA-48_	*bla *_NDM_, _VIM_	*bla *_OXA-51_, _OXA-21_,_OXA-58_, _IMP_	*bla *_mecA_	*bla *_VanA_
Initial denaturation	94°C/5 min	94°C/5 min	94°C/10 min	94°C/10 min	95°C/2 min	95°C/1 min	94°C/2 min
Denaturation	94°C/1 min	94°C/1 min	94°C/30s	94°C/30 s	95°C/5 s	94°C/1 min	94°C/1 min
Annealing	60°C/1 min	42°C/1 min	55°C/1 min 30s	52°C/1 min 30s	60°C/10 s	52°C/1 min	54°C/1 min
Extension	72°C/1 min	72°C/1 min	72°C/1 min 30s	72°C/1 min 30s	72°C/10 s	72°C/1 min	72°C/1 min
Final elongation	72°C/10 min	72°C/10 min	72°C/10 min	72°C/10 min	72°C/10 s	72°C/10 min	72°C/10 min
Number of cycles	30	35	35	35	40	35	35

**Table 4:** Yields of *C. salviifolius* and *C. monspeliensis* extractions.

*Cistus* species	Solvent used	Yield with maceration (%)	Extracts ID	Yield with Soxhlet (%)	Extracts ID
*C. monspeliensis*	Methanol	28.8	CCMM	56.66	CCMS
	Hexane	1.5	F1CMM	1.2	F1CMS
	Dichloromethane	4.74	F2CMM	8.4	F2CMS
	Ethyl acetate	2.25	F3CMM	12	F3CMS
	*n*-butanol	6.25	F4CMM	16	F4CMS
	Remaining aqueous	13	F5CMM	18.8	F5CMS

*C. salviifolius*	Methanol	27.9	CCSM	31	CCSS
	Hexane	0.7	F1CSM	1	F1CSS
	Dichloromethane	0.7	F2CSM	1.33	F2CSS
	Ethyl acetate	1	F3CSM	3.66	F3CSS
	*n*-butanol	7	F4CSM	3.33	F4CSS
	Remaining aqueous	16.7	F5CSM	20.3	F5CSS

CCMM: crude CM from maceration; CCMS: crude CM from Soxhlet; F1CMM: hexane fraction of CM from maceration; F1CMS: hexane fraction of CM from Soxhlet; F2CMM: dichloromethane fraction of CM from maceration; F2CMS: dichloromethane fraction of CM from Soxhlet; F3CMM: ethyl acetate fraction of CM from maceration; F3CMS: ethyl acetate fraction of CM from Soxhlet; F4CMM: *n*-butanol fraction of CM from maceration; F4CMS: *n*-butanol fraction of CM from Soxhlet; F5CMM: remaining aqueous of CM from maceration; F5CMS: remaining aqueous of CM from Soxhlet; CCSM: crude CS from maceration; CCSS: crude CS from Soxhlet; F1CSM: hexane fraction of CS from maceration; F1CSS: hexane fraction of CS from Soxhlet; F2CSM: dichloromethane fraction of CS from maceration; F2CSS: dichloromethane fraction of CS from Soxhlet; F3CSM: ethyl acetate fraction of CS from maceration; F3CSS: ethyl acetate fraction of CS from Soxhlet; F4CSM: *n*-butanol fraction of CS from maceration; F4CSS: *n*-butanol fraction of CS from Soxhlet; F5CSM: remaining aqueous fraction of CS from maceration; F5CSS: remaining aqueous fraction of CS from Soxhlet.

**Table 5:** Phytochemical composition of ethyl acetate and *n*-butanol fractions from two *Cistus* species.

Identified compound	Area (%)			
	*C. monspeliensis*		*C. salviifolius*	
	Ethyl acetate	*n*-butanol	Ethyl acetate	*n*-butanol
**Monoterpene hydrocarbons**	**21.15**	**20.23**	**3.8**	**16.93**
*α*-Pinene	2.26	2.74	0.36	1.97
*β*-Pinene	1.85	—	—	—
*β*-Myrcene	1.06	8.22	1.46	1.28
Camphene	1.36	1.37	0.21	1.01
*α*-Phellandrene	6.70	3.24	—	5.97
Limonene	7.92	4.66	1.77	6.70
**Monoterpenes oxygenated**	**1.64**	**2.26**		**5.4**
Eucalyptol (1,8-cineol)	1.64	2.26	—	1.84
**Sesquiterpenes hydrocarbons**	**3.89**			
Cedrene	3.89	—	—	—
**Sesquiterpenes oxygenated**	**61.41**	**5.34**	**70.77**	**9.41**
Viridiflorol	59.84	—	70.77	6.92
Elemol	—	5.43	—	—
Agarospirol	—	—	—	2.49
2,6-Di-tert-butyl-p-cresol	1.57	—	—	—
**Phenolic compounds**	**1.01**	**5.77**		**5**
2,4-Bis(1,1-dimethylethyl)-phenol	1.01	5.77	—	5.00
**Nonterpene compounds**	**10.9**	**66.31**	**3.90**	**63.26**
Hydrocarbons		**36.81**		**44.75**
7,9-Dimethyl-hexadecane	—	6.72	—	7.59
5,7-Dimethyl-undecane	—	1.39	—	3.65
8-Hexyl-pentadecane	—	—	—	6.51
5-(2-Methylpropyl)-nonane	—	2.18	—	—
2-Methyl-eicosane	—	2.24	—	—
2,6,10,15-Tetramethyl-heptadecane	—	—	—	2.94
8-Methyl-heptadecane	—	—	—	8.08
10-Methyl-eicosane	—	6.00	—	4.61
7-Hexyl-eicosane	—	—	—	1.77
Eicosane	—	1.46	—	1.51
2,6,11,15-Tetramethyl-hexadecane	—	5.76	—	—
2,4-Dimethyl-undecane	—	5.61	—	—
2,6,10,14,18-Pentamethyl-eicosane	—	5.45	—	8.09
Alcohols	**2.73**	**6.74**		**4.48**
2-(2-Hydroxypropoxy)-1-propanol	2.73	—	—	2.00
2-Isopropyl-5-methyl-1-heptanol	—	2.02	—	—
2-Ethyl-2-methyl-tridecanol	—	4.72	—	2.48
Carboxylic acids	**4.33**		**3.90**	
4-Acetyl benzoic acid	4.33	—	3.90	—
Fatty acids	**1.22**			
Pelargonic acid	1.22	—	—	—
Alkanes	**2.62**			
1,1′-Oxybis-2-propanol	2.62	—	—	—
1,1-Dibutoxy-butane	—	—	—	3.56
Ketones		**2.17**		**0.87**
2-Heptyl-4-methyl-1,3-dioxane	—	2.17	—	0.87
Ester		**9.39**		**13.16**
Butyl butyrate	—	9.39	—	13.16
Ether		**3.83**		
Butane, 1,1-dibutoxy-Heptadecane, 8-methyl-	—	3.83	—	—
Unknown	—	7.37	—	—

**Table 6:** Antibiotic susceptibility testing and screening of gene-mediated resistance to antibiotics by PCR.

Strains	Antibiotic	Antibiogram	Drug resistance gene
*K. pneumoniae*	AP	R	*bla *_SHV_ *bla *_OXA-48_
	AMC	R	
	CFX	R	
	CRO	S	
	CTX	S	
	MEM	R	
	ETP	R	
	TN	R	
	CIP	R	
	TS	R	
	CL	S	

*E. cloacae*	AP	R	*bla *_CTX-M_ *bla *_OXA-48_
	AMC	R	
	CFX	R	
	CRO	R	
	CTX	R	
	MEM	S	
	ETP	R	
	TN	R	
	CIP	R	
	TS	R	
	CL	S	

*A. baumannii*	IMP	R	*bla *_OXA-51_ *bla *_OXA-58_ *bla *_IMP_ *bla *_VIM_
	MEM	R	
	GM	R	
	NET	S	
	AK	R	
	TSU	R	
	CIP	R	
	LEV	R	
	TN	R	
	CL	R	

*P. aeruginosa*	CAZ	R	*bla *_NDM_
	IMP	R	
	GM	R	
	AK	R	
	NET	R	
	CIP	S	
	PTZ	R	
	CPM	R	
	LEV	R	
	TN	R	
	CL	S	

*S. aureus*	PG	R	*bla *_mecA_
	GM	S	
	TN	R	
	K	R	
	CIP	I	
	TSU	R	
	FOX	R	
	E	S	

*E. faecium*	AMP	R	*bla *_VanA_
	TSU	R	
	GM	R	
	CIP	R	
	LEV	R	
	LNZ	S	
	VA	R	
	TEC	R	

S: susceptible; R: resistant.

**Table 7:** Determination of IZD (mm), MIC, and MBC (mg/mL) of *C. monspeliensis* extracts.

Strains	*E. faecium*				*S. aureus*				*K. pneumoniae*				*A. baumannii*				*P. aeruginosa*				*E. cloacae*			
Extracts	IZD mm	MIC mg/mL	MBC	MBC/MIC	IZD	MIC	MBC	MBC/MIC	IZD	MIC	MBC	MBC/MIC	IZD	MIC	MBC	MBC/MIC	IZD	MIC	MBC	MBC/MIC	IZD	MIC	MBC	MBC/MIC
CMM	17	0.42	0.85	2	20	0.01	0.42	32	12	1.75	6.87	4	15	0.10	3.43	32	12	0.21	6.87	33	14	0.85	27.5	32
CMS	13	0.42	0.42	1	14	0.05	0.85	16	9	13.75	13.75	1	12	0.42	0.42	1	10	0.42	6.87	6	10	3.43	13.75	4
F1MM	11	0.42	1.71	4	10	0.01	0.05	4	10	6.87	13.75	2	9	3.43	3.43	1	10	3.42	13.75	4	8	3.43	6.87	2
F1MS	10	0.42	0.42	1	10	0.01	1.71	131	9	6.87	13.75	2	8	3.43	6.87	2	10	3.43	13.75	4	8	6.87	13.75	2
F2MM	11	0.10	0.42	4	11	0.01	0.10	8	10	3.43	6.87	2	10	3.43	6.87	2	9	1.71	6.87	4	10	3.43	13.75	4
F2MS	10	0.21	1.71	8	10	0.02	0.85	32	9	6.87	6.87	1	6	3.43	6.87	2	7	3.43	6.87	2	9	3.43	27.5	8
F3MM	17	0.85	1.71	2	17	0.01	0.10	8	13	0.42	6.87	16	15	0.85	3.43	4	15	0.02	6.87	33	14	3.43	13.75	4
F3MS	14	0.42	0.42	1	13	0.01	0.85	65	10	3.43	6.87	2	13	0.05	0.85	16	13	0.02	3.43	131	11	3.43	13.75	4
F4MM	14	0.85	6.87	8	13	0.01	0.85	65	9	0.85	13.75	16	10	1.71	6.87	4	11	3.43	6.87	2	10	6.87	27.5	2
F4MS	11	0.85	0.85	1	10	0.10	3.43	32	7	0.85	6.87	8	7	1.71	3.43	2	9	6.87	6.87	1	8	6.87	27.5	2
F5MM	13	0.85	1.71	2	13	0.05	0.85	16	11	3.43	6.87	2	10	1.71	6.87	4	12	0.10	6.87	64	10	6.87	13.75	2
F5MS	11	0.85	1.71	2	10	0.21	1.71	8	8	6.87	13.75	2	7	3.43	3.43	1	6	1.71	6.87	4	8	6.87	27.5	4

CCMM: crude CM from maceration; CCMS: crude CM from Soxhlet; F1CMM: hexane fraction of CM from maceration; F1CMS: hexane fraction of CM from Soxhlet; F2CMM: dichloromethane fraction of CM from maceration; F2CMS: dichloromethane fraction of CM from Soxhlet; F3CMM: ethyl acetate fraction of CM from maceration; F3CMS: ethyl acetate fraction of CM from Soxhlet; F4CMM: *n*-butanol fraction of CM from maceration; F4CMS: *n*-butanol fraction of CM from Soxhlet; F5CMM: remaining aqueous of CM from maceration; F5CMS: remaining aqueous of CM from Soxhlet.

**Table 8:** Determination of IZD (mm), MIC, and MBC (mg/mL) of *C. salviifolius* extracts.

Strains	*E. faecium*				*S. aureus*				*K. pneumoniae*				*A. baumannii*				*P. aeruginosa*				*E. cloacae*			
Extracts	IZD mm	MIC mg/Ml	MBC	MBC/MIC	IZD	MIC	MBC	MBC/MIC	IZD	MIC	MBC	MBC/MIC	IZD	MIC	MBC	MBC/MIC	IZD	MIC	MBC	MBC/MIC	IZD	MIC	MBC	MBC/MIC
CSM	15	0.21	1.71	8	16	0.10	0.42	4	7	3.43	13.75	4	8	0.85	3.43	4	9	0.05	3.43	64	8	1.71	13.75	8
CSS	13	0.42	0.42	1	14	0.10	3.43	32	6	6.87	6.87	1	6	0.21	1.71	8	7	0.10	6.87	64	6	3.43	6.87	2
F1SM	10	3.43	6.87	2	12	0.01	0.85	65	8	6.87	13.75	2	7	3.43	6.87	2	8	0.85	13.75	16	9	6.87	6.87	1
F1SS	10	0.42	1.71	4	12	0.10	3.43	32	8	6.87	13.75	2	6	3.43	6.87	2	7	0.05	13.75	259	8	3.43	13.75	4
F2SM	13	1.87	6.87	4	14	0.01	0.21	16	9	3.43	27.5	8	8	1.71	3.43	2	8	1.71	6.87	4	9	3.43	13.75	4
F2SS	11	3.43	6.87	2	12	0.85	6.87	8	7	13.75	13.75	1	6	3.43	3.43	1	7	3.43	13.75	4	7	3.43	27.5	8
F3SM	14	0.42	0.85	2	14	0.01	0.05	4	12	0.42	6.87	16	12	3.43	3.43	1	13	1.71	3.43	2	12	3.43	6.87	2
F3SS	12	0.21	0.21	1	12	0.10	3.43	32	9	3.43	6.87	2	6	1.71	1.71	1	13	3.43	3.43	1	9	6.87	13.75	2
F4SM	14	0.42	0.85	2	15	0.01	0.21	16	10	0.42	6.87	16	12	0.85	3.43	4	13	0.05	6.87	129	10	6.87	13.75	2
F4SS	11	1.71	3.43	2	12	0.26	6.87	26	8	6.87	6.87	1	6	1.71	3.43	2	9	3.43	13.75	4	7	6.87	27.5	4
F5SM	15	0.85	1.71	2	16	0.05	0.21	4	11	3.43	6.87	2	13	1.71	6.87	4	15	0.02	6.87	264	10	6.87	13.75	2
F5SS	11	6.87	6.87	1	12	3.43	13.75	4	8	6.87	6.87	1	6	1.71	1.71	1	12	3.43	13.75	4	7	6.87	27.5	2

CCSM: crude CS from maceration; CCSS: crude CS from Soxhlet; F1CSM: hexane fraction of CS from maceration; F1CSS: hexane fraction of CS from Soxhlet; F2CSM: dichloromethane fraction of CS from maceration; F2CSS: dichloromethane fraction of CS from Soxhlet; F3CSM: ethyl acetate fraction of CS from maceration; F3CSS: ethyl acetate fraction of CS from Soxhlet; F4CSM: *n*-butanol fraction of CS from maceration; F4CSS: *n*-butanol fraction of CS from Soxhlet; F5CSM: remaining aqueous fraction of CS from maceration; and F5CSS: remaining aqueous fraction of CS from Soxhlet.

## Data Availability

All data used to support the findings of this study are included in this article.
